# Cooperate or not cooperate EEG, autonomic, and behavioral correlates of ineffective joint strategies

**DOI:** 10.1002/brb3.902

**Published:** 2018-01-05

**Authors:** Michela Balconi, Laura Gatti, Maria Elide Vanutelli

**Affiliations:** ^1^ Research Unit in Affective and Social Neuroscience Catholic University of Milan Milan Italy; ^2^ Department of Psychology Catholic University of Milan Milan Italy

**Keywords:** cooperation, electroencephalographic, frustration, negative feedback, skin conductance activity, strategies

## Abstract

**Introduction:**

The neural activity in response to ineffective joint actions was explored in the present study. Subjects involved in a cooperative but frustrating task (poor performance as manipulated by an external feedback) were required to cooperate (T1) during an attentional task in a way to synchronize their responses and obtain better outcomes.

**Methods:**

We manipulated their strategies by providing false feedbacks (T2) signaling the incapacity to create a synergy, which was reinforced by a general negative evaluation halfway through the game. A control condition was provided (no cooperation required, T0) as well as a check for possible learning effect (time series analysis). The effects of the feedback in modulating subjects' behavioral performance and electrocortical activity were explored by means of brain oscillations (delta, theta, alpha, beta) and autonomic activity (heart rate, HR; skin conductance activity, SCR).

**Results:**

Results showed a specific pattern of behavioral, neural, and peripheral responses after the social feedback. In fact, within this condition, worse behavioral outcomes emerged, with longer response times with respect to the prefeedback one. In parallel, a specific right‐lateralized effect was observed over the dorsolateral prefrontal cortex (DLPFC), with increased delta and theta power compared to the previous condition. Moreover, increased SCR was observed with respect to the first part.

**Conclusions:**

Two interpretations are put forward to explain the present findings: 1) the contribution of negative emotions in response to failing interactions or 2) a motivational disengagement toward goal‐oriented cooperation elicited by frustrating evaluations.

## 1 INTRODUCTION

The term cooperation refers to collaborative actions involving two or more individuals finalized to obtain common behavioral effects. This kind of behavior is planned, acted, and directed toward a specific goal or the fulfillment of actions which imply common interests. Also, it generally secures a benefit to all the actors involved. As a possible mediator of such processes, the capacity to perceive and infer others' affective states could be pivotal, from more basic resonance and mirroring abilities, toward the development of complex social exchange based on joint attention and synchronization (Baker et al., 2016; Balconi & Bortolotti, 2012; Liu, Saito, & Oi, 2015; Vanutelli, Nandrino, & Balconi, 2016).

In parallel to synchronized behavioral effects, cooperative performances during an interpersonal task principally involve a process of social cognition. Previous research explored the effect of cooperation on self‐perceived efficacy in social interactions and cognition within social hierarchies. Such studies showed that a cooperative condition may reinforce the sense of membership to a group. Also, it may increase the sense of self‐efficacy, a general social well‐being, interpersonal relationships, and the perception of higher social positions (Balconi & Pagani, 2015; Chung, Yun, & Jeong, 2015; Cui, Bryant, & Reiss, 2012; Funane et al., 2011; Goldman, Stockbauer, & McAuliffe, 1977).

Recent research examined the structure and function of brain areas associated with social perception, interactions, and cooperation efficacy. Specifically, previous studies explored the effect of positive outcomes on self‐perception (Balconi & Pagani, 2014; Balconi & Vanutelli, 2017a; Bouffard‐Bouchard, 1990), performance (Balconi & Pagani, 2014, 2015; Balconi & Vanutelli, 2017a; Locke, Frederick, Lee, & Bobko, 1984), and brain responsiveness during cooperative or competitive tasks with respect to interpersonal feedbacks (Balconi & Vanutelli, 2016, 2017a).

Results suggested the contribution of prefrontal neural mechanism in response to cooperative tasks (Baker et al., 2016; Cui et al., 2012; Liu et al., 2015; Suzuki, Niki, Fujisaki, & Akiyama, 2011). Indeed, it was observed that specific neural networks linking limbic regions and the prefrontal cortex (PFC) may support the affective, cognitive, and behavioral components of social interactions during cooperation (Levitan, Hasey, & Sloman, 2000). Specifically, it was found that the dorsal (DLPFC) and ventral (VLPFC) portions of the lateral PFC are generally engaged during inferences about social status (Balconi & Pagani, 2014, 2015; Chiao et al., 2009). The activation of these areas during social interactions that involve ranking perception probably highlights the recruitment of top‐down control mechanisms over specific emotional responses to social events, in a way to plan appropriate reactions (Marsh, Blair, Jones, Soliman, & Blair, 2009). In fact, these brain regions are typically involved in the regulation of socio‐emotional responses and behavioral inhibition.

However, in some cases, this positive effect is disrupted due to the perception of ineffective outcomes of our own actions. Indeed, an important construct that can be used to mediate the brain responsiveness is the perception of effective versus ineffective interactions. In fact, this feedback can be considered a powerful cue that can reciprocally reinforce or weaken behavior toward a common goal and a relevant tool to train the brain to work jointly. Therefore, what could we expect when cooperation is ineffective? Different possible scenarios may be suggested when an unsuccessful cooperation is self‐represented. Firstly, a more competitive behavior may be adopted, simulating a “dysfunctional” interaction with a consequent “disengaged” relation, as, in the absence of a proficient cooperation, the synergic plan could be disrupted. Indeed, depending on the interaction modalities (positive or negative cooperation), individuals may either facilitate or obstruct others' goal achievement and self‐represent themselves as more or less proficient in relation to others. Some previous work demonstrated that one's own actions are facilitated when perceiving others' ones as effective (Knoblich & Jordan, 2003; Sebanz, Knoblich, & Prinz, 2003). In contrast, in the case of competition or ineffective interactions, the other agent's behavior is less predictable than that in the case of cooperation, in which there is a planned expectation. At this regard, we probably need to recalibrate the mental representation and to modify previous cognitive plans. As such, this condition imposes an increase in cognitive load. Similarly, an unsuccessful strategy, although in a cooperative context, may require supplementary cognitive resources to update and modify the joint action. These mechanisms rely on executive functions and, specifically, on the selection of salient knowledge or response to achieve new internally represented goals and strategies (Humphrey, 1988; Leslie, 1987). In this perspective, the strong increase in prefrontal cortex activity—mainly the medial prefrontal cortex—observed during competition or in the case of a failure may in part mirror higher executive processing demands (Decety, Jackson, Sommerville, Chaminade, & Meltzoff, 2004).

A second possibility to predict behavioral and brain response in the case of failure could be more directly related to the emotional impact of an ineffective cooperation, where subjects may develop negative and withdrawal emotions toward their own partner due to the inefficacy of the joint action. This should involve some more prefrontal lateralized areas related to the effect of an emotional empathic response. That is the negative emotional behavior may be considered as a “safeguard” produced by the need of reparative strategies, to compensate the reciprocal inefficacy and to try to reach a more proficient common strategy (Balconi, Bortolotti, & Gonzaga, 2011; Balconi & Canavesio, 2013, 2014). In this case, based on the valence model of emotions, the lateralization effect could suggest a more right prefrontal unbalance that was found to support more negative or avoidant emotional contexts (Balconi & Canavesio, 2014; Balconi, Grippa, & Vanutelli, 2015; Davidson, 1998; Morinaga et al., 2007; Tuscan et al., 2013).

Therefore, in this study, the cortical response to this particular condition was explored using behavioral, electroencephalographic (EEG), and autonomic (by biofeedback device) measures to test the role of prefrontal lateralization effect, and, more generally, the role of emotions and the cognitive impact of an ineffective cooperation. No previous research monitored these three components all together to furnish a complete analysis of the emotional impact in the case of dysfunctional cooperation.

On the one hand, brain oscillations may be considered as a valid measure of brain activation, as they have often been applied to describe distinct responsiveness by the two hemispheres to different emotional and social conditions (Balconi, Falbo, & Conte, 2012; Balconi & Mazza, 2009; Balconi & Vanutelli, 2015; Sutton & Davidson, 1997). Indeed, EEG modulation was used to demonstrate the lateralized PFC responsiveness related to emotional processing. Indeed, in previous studies, a reduction in alpha power (increased cortical activity) in left frontal areas was found in response to approach attitude (Balconi, Brambilla, & Falbo, 2009a,b; Balconi & Mazza, 2010; Balconi et al., 2011; Davidson, 1992, 2004; Harmon‐Jones, 2004).

For what concerns other frequency bands, their role in emotion processing is less defined: Some studies showed that theta band power responds to emotional stimulation (Knyazev, 2007; Krause, Enticott, Zangen, & Fitzgerald, 2012) in response to coordinated response to alertness and readiness (Balconi et al., 2009a; Başar, 1999), and this is of particular importance if we consider that neurons in the amygdala produce theta activity during emotional arousal (Başar, 1999; Bekkedal, Rossi, & Panksepp, 2011; Paré, 2003).

About delta band, Knyazev (2007) reported that it is related to motivational systems and salience detection. In addition, both delta and theta modulations were found to be associated with the arousing power of the stimuli in the right and left frontal localizations. Also, an increase in theta and delta frequency bands was found during negative‐valenced emotional stimulation in healthy adults (Balconi et al., 2015). Therefore, these low‐frequency bands proved to be related to motivational and attentional significance of salient affective stimuli (Balconi & Pozzoli, 2009; Balconi et al., 2009a; Başar, 1999).

Focusing more directly on interactive studies, Sänger, Müller, and Lindenberger (2012) found, in dyads of guitarists playing together, that delta and theta phase locking were enhanced at frontal and central electrodes during phases that required high demands on musical coordination. Considering high‐frequency activities, instead, higher beta and gamma responses were found in prefrontal regions during cooperative decisions taken together in a task involving incentives (Chung, Yun, & Jeong, 2008). For what concerns competition, instead, Babiloni and colleagues (Babiloni et al., 2007), in a task simulating a card game, found a larger activity in prefrontal and anterior cingulated cortex within different frequency bands in the player that leaded the game, if compared to other players.

In parallel with EEG recording, autonomic indices were considered potential markers of emotional condition (Tupak et al., 2014). The acquisition of both central and peripheral measures has the advantage of better elucidating the reciprocal interplay of the two measures. Among the others, skin conductance response (SCR) offers a useful measure of the limbic function (Furmark, Fischer, Wik, Larsson, & Fredrikson, 1997; Lang, Davis, & Ohman, 2000). The significance of this measure for emotion and arousal modulation was previously demonstrated (Balconi & Bortolotti, 2014; Balconi & Pozzoli, 2008; Balconi et al., 2009b, 2015). Also several EEG studies revealed a direct relation between PFC activation and the autonomic nervous system in response to emotional stimulation.

For example, Tanida and colleagues (Tanida, Katsuyama, & Sakatani, 2007) reported that the degree of right‐lateralized asymmetry in PFC activation during mental stress was positively correlated with the degree of activation of the sympathetic nervous system. These studies offer partial support to the role of PFC in processing visceral reactions, or somatic markers, associated with social–emotional condition.

Therefore, we planned a specific paradigm which monitored the negative feedback effect (of failure) on behavioral, central (EEG), and autonomic (by biofeedback device) components when cooperation goes wrong. It was done to explore the modulation of the joint strategy and the emotional impact on the behavioral and brain activity. A control condition (absence of a cooperative task) was included to compare the effect of cooperation and joint action with individual performance without a cooperative task. Based on previous hypotheses, the postfeedback condition (artificially ineffective performance) could show one of these scenarios: As found in previous research on competition, a specific generalized increased prefrontal activity is attended in order to manage an unexpected and more complex situation (failure), when subjects realize they are not efficient in synchronizing their actions. In contrast, a more emotionally directed perspective foresees the implication of different and selective areas of the PFC, with a specific lateralization effect within the right hemisphere in response to a significant negative emotional effect, related to a social situation perceived as frustrating and uncertain from a relational point of view. In this last case, the more arousing condition should significantly modulate the autonomic behavior with a general higher HR and SCR. Also, the behavioral performance should be affected by negative feedback, with an increased cognitive difficulty to manage the synchronized strategy.

## 2 MATERIALS AND METHODS

### 2.1 Participants

Twenty undergraduate students (*M* = 22.32, *SD* = 1.93; male = 9) took part in the experiment. The participants were all right‐handed and presented normal or corrected‐to‐normal visual acuity. Exclusion criteria were history of psychopathology (Beck Depression Inventory, BDI‐II, Beck, Steer, & Brown, 1996) for the subjects and immediate family. Also, State‐Trait Anxiety Inventory (STAI, Spielberger, Gorsuch, Lushene, Vagg, & Jacobs, 1970) was submitted after the experimental session. Based on a clinical screening, no neurological or psychiatric pathologies were observed. No form of dependence (alcohol or drug abuse or addiction) was observed by a specific screening, and consume of alcoholic or energetic drinks in the period before the experiment was discouraged and controlled. The experimental dyad was composed by one male and one female, and the participants did not met before each other. No payment was provided for subjects' participation. They all gave informed written consent to participate in the study. Finally, the research was conducted in accordance with the Declaration of Helsinki, and it was approved by the local ethics committee of the Department of Psychology, Catholic University of Milan.

### 2.2 Procedure

Subjects were comfortably seated in a darkened room with a pc screen placed approximately 60 cm in front of their eyes. The dyads were seated side by side and were divided by a black screen to prevent visual or physical contact. They performed a simple task of sustained selective attention. Subjects were told that some attentional measures would have been used to assess their subjective skills during cooperation (t1) and, to enhance their motivation, that these measures are usually used as a screening in the workplace to test professional career success and teamwork capabilities. Thus, the cooperative nature of the task was stressed: Participants were told that their scoring was based on the ability to synchronize their responses, in terms of both accuracy (number of correct responses: hits) and response times (RTs), with another partner.

Participants were required to memorize and, then, recognize simple geometric figures (targets) among distractors by making a two‐alternative forced‐choice with left/right buttons. Target's features changed every 25 trials; they were displayed for 500 ms and separated by a 300‐ms interstimulus interval (ISI).

The task was a modified version of previous experimental paradigms involving competitive instructions (Balconi & Vanutelli, 2016, 2017b) or cooperative dynamics with a good outcome (Balconi & Vanutelli, 2017a). The present version presents two main variations: First, cooperative strategies were frustrated by giving subjects negative feedbacks about their performance. In fact, halfway through the task, participants received a general evaluation about their joint performance which was manipulated a priori, and were told they had a bad cooperation (synchronicity) score with 26% in terms of speed synchrony, and 31% in terms of accuracy synchrony. They were also encouraged to change and improve their performance score during the second part of the experiment.

**Figure 1:**
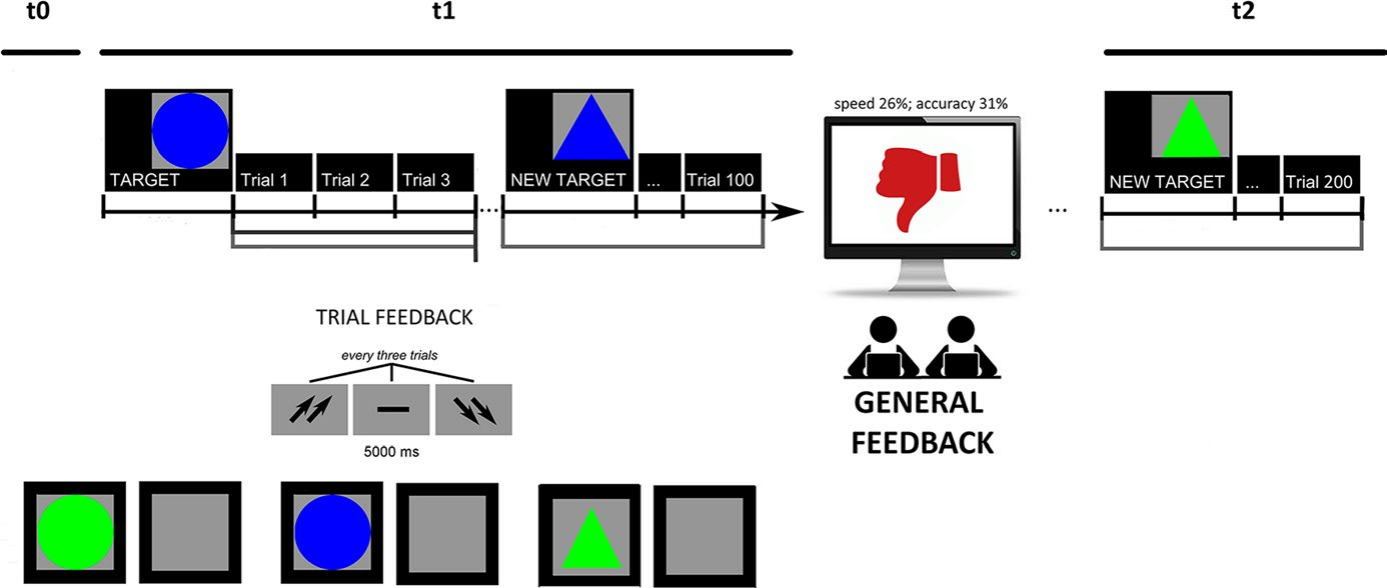
Experimental procedure which represents the setting, the attentional task, and EEG/autonomic activity recording

Secondly, this task was composed by three sessions: a first preliminary phase (control condition) where subjects were not asked to cooperate, but only to execute the attention task individually (t0); then, a second phase (t1) where subjects were required to synchronize their performance (four blocks before the feedback, 100 trials); and a third phase (t2), which followed the negative social feedback described above (four blocks after the feedback, 100 trials) (Figure 1).

To develop shared cooperative strategies in t1 and t2, participants were told that they would have systematically received a feedback in response to each trial (trial feedback), which was composed by three stimuli. The feedback was signaled by two up arrows (high cooperation score), a dash (mean performance), or two down arrows (low cooperation score), and was displayed on the screen for 5000 msec. After that, an intertrial interval (ITI) occurred and lasted for other 5000 ms. Across the task, after the initial mean performance, subjects were regularly informed about their performance by presenting the down arrows in 70% of cases, while the dash or the up arrows appeared in 30% of cases.

Moreover, subjects were required to evaluate their performance and motivation efficacy on a 7‐point Likert scale (from 1 = no agreement to 7 = high agreement) in terms of the importance they attributed to the social context and the feedback of the task; their trust on the feedback received about their performance; and finally the relevance of this feedback to represent their social status and social position. Based on this postexperiment questionnaire, participants were strongly engaged in the hierarchical context (they reported to be highly engaged, *M* = 5.98; *SD* = 0.45). The subjects were also required to self‐report their degree of trust in the exact feedback of the performance, which showed high trust (*M* = 6.34; *SD* = 0.33) and the relevance of the task for the social status (*M* = 5.90; *SD* = 0.48).

### 2.3 Performance scoring

The cognitive performance was measured by considering reaction times (RTs, msec, recorded from the stimulus onset) and the error rates (ERs, calculated as the total number of incorrect detections out of the total trial) for each category.

### 2.4 EEG recording and analysis

EEG recordings were performed with two 16‐channel EEG systems (V‐AMP: Brain Products, München. Truscan: Deymed Diagnostic, Hronov) with electrodes positioned over AFF1 h, Fz, AFF2 h, FFC3 h, FFC4 h, C3, Cz, C4, P3, Pz, P4, O1, O2, T7, and T8. An ElectroCap with Ag/AgCl electrodes was used to record EEGs from active scalp sites referred to the earlobes (10/5 international system; Oostenveld & Praamstra, 2001). Data were acquired using a sampling rate of 500 Hz, with a frequency band of 0.01 to 40 Hz. An offline common average reference was successively computed to limit the problems associated with the signal‐to‐noise ratio (Ludwig et al., 2009). One EOG electrode was placed on the outer canthi to detect eye movements. The impedance of the recording electrodes was monitored for each subject prior to data collection and was always below 5 kΩ. The signal was visually scored, and portion of the data that contained artifacts was removed to increase specificity. Blinks were also visually monitored. Ocular artifacts (eye movements and blinks) were corrected using an eye movement correction algorithm that employs a regression analysis in combination with artifact averaging (Sapolsky, 2004). In addition, a standard ICA analysis was applied (Jung et al., 2000). After performing EOG correction and visual inspection, only artifact‐free trials were considered (rejected epochs, 1%).

The digital EEG data were bandpass‐filtered offline (0.1–40 Hz, 48 dB/octave roll‐off), and frequency power data were computed by fast Fourier transformation (FFT) for standard frequency bands: delta (0.5–4 Hz), theta (4–8 Hz), alpha (8–12 Hz), and beta (14–20 Hz). An individual average power value for each experimental condition and for baseline recordings was calculated for each EEG channel. This method is often used in paradigms where stimuli are continuously repeated at a fixed frequency for extended time periods (Roach & Mathalon, 2008). Also, to obtain a signal proportional to the power of the EEG frequency band, the filtered signal samples (epoch 1000 ms) were squared, thus resulting in the total power of the EEG at each frequency, time point (Roach & Mathalon, 2008), and channel.

**Figure 2:**
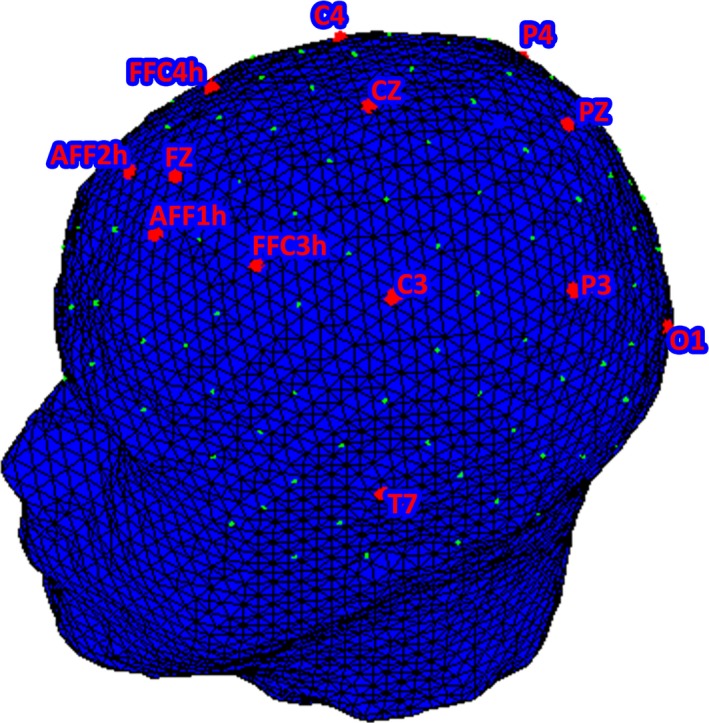
EEG montage over AFF1 h, Fz, AFF2 h, FFC3 h, FFC4 h, C3, Cz, C4, P3, Pz, P4, O1, O2, T7, and T8

Considering the statistical analyses, only the lateralized activity over anterior frontal (AFF1 h, AFF2 h), frontal (FFC3 h, FFC4 h), central (C3, C4), and parietal (P3, P4) electrodes was considered (Figure 2).

### 2.5 Autonomic measures

A biofeedback device (Biofeedback 2000, version 7.01) connected to a personal computer was used only to record autonomic activity and was not used to provide feedbacks to the subjects. One set of electrodes was connected to the Biofeedback Amplifier. To measure SCR (electrodermal activity or the electrical conductance of the skin), the skin was cleaned with alcohol and slightly abraded before attaching the electrodes. The electrodes (4 mm diameter Ag/AgCl electrodes), filled with Surgicon electrolyte paste, were positioned over the medial phalanges of the second and third fingers of the nondominant hand (Amrhein, Mühlberger, Pauli, & Wiedemann, 2004). SCR elicited by each stimulus was registered continuously with a constant voltage. It was manually scored and defined as the largest increase in conductance during the task, with a cutoff of at least 0.4 μS in amplitude with respect to prestimulus mean values. Prestimulus values were scored during the 5 s. prior to stimulus onset. The electrocardiogram was recorded using electrodes on the left and right forearms. Interbeat intervals of the electrocardiogram were converted to heart rate (HR) in number of beats per minute (scoring HR modulation while viewing emotional cues). Trials with artefacts were excluded from analysis, whereas trials with no detectable response were scored as zero.

## 3 RESULTS

A preliminary analysis was applied to t0 (no cooperative task) compared to t1 (prefeedback cooperative task) and t2 (postfeedback cooperative task). Both the components of the dyad were included in the analysis. Systematic significant differences were found between t0 versus t1 and t2, for both behavioral and neurophysiological measures. These results support the specificity of cooperative contexts compared to the absence of cooperation task.

We reported the main effects we found between t0 versus t1 and t0 versus t2 (Table 1) in the preliminary phase of analysis. It was done in order to preliminary compare the effect due to the cooperation versus the absence of cooperation condition.

**Table 1:** Mean(standard deviations) for each measure (behavioral; EEG: autonomic) as a function of condition (t0; t1; t2)

	T0	T1	T2
Behavioral measures			
ERs	0.15 (0.007)	0.10 (0.004)	0.09 (0.008)
RTs	298 (16)	314 (12)	379 (20)
EEG measures			
delta	4.12 (0.21)	5.75 (0.14)	6.76 (0.17)
theta	4.02 (0.17)	5.09 (0.13)	5.69 (0.19)
alpha	5.98 (0.21)	5.41 (0.17)	4.56 (0.18)
beta	4.87 (0.17)	5.09 (0.24)	5.25 (0.20)
Autonomic measures			
SCR	1.96 (0.03)	2.11 (0.03)	3.44 (0.01)
HR	74 (0.13)	79 (0.23)	82 (0.29)

EEG, electroencephalographic; ERs, error rates; RTs, response times.

Therefore, we considered in the second phase the direct comparison between t1 and t2, to focus on the feedback effect. Three sets of analyses were performed with respect to behavioral (ERs; RTs) and neurophysiological (EEG and autonomic) measures. A first set of repeated measures ANOVAs with independent factor condition (Cond: pre vs. post feedback) was applied to ER and RTs. The same analysis design was applied to the autonomic‐dependent variables (HR; SCR).

In the case of EEG measure, repeated measures ANOVAs with Cond, localization (Loc: anterior frontal, DLPFC, central, parietal), and lateralization (Lat: left vs. right) as independent factor were applied to each frequency band.

For all of the ANOVA tests, the degrees of freedom were corrected using Greenhouse–Geisser epsilon where appropriate. Post hoc comparisons (contrast analyses) were applied to the data. Bonferroni test was applied to multiple comparisons. In addition, the normality of the data distribution was preliminary tested (kurtosis and asymmetry tests). The normality assumption of the distribution was supported by these preliminary tests.

To exclude a possible learning effect, a preliminary analysis was applied, comparing separately the first set of four intervals (before feedback) and the second set four intervals (post feedback) in all the dependent measures (RTs, ERs, EEG, autonomic). As no significant differences among the four intervals, respectively, before and after the feedback were found, we did not include this factor in the successive analysis.

### 3.1 RTs and ERs

**Figure 3:**
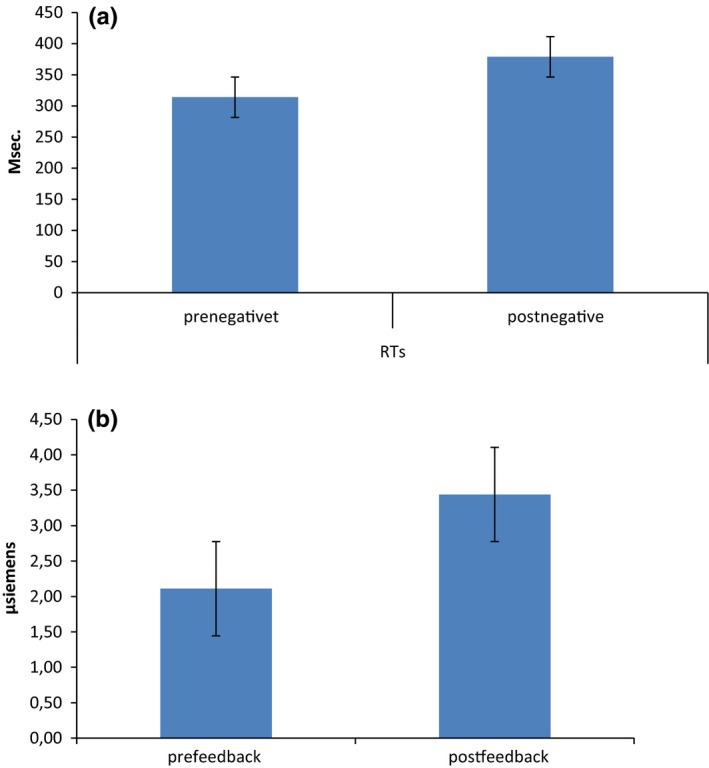
(a) RTs modulation as a function of pre‐ and postfeedback conditions. The postfeedback condition was characterized by longer RTs. (b) SCR modulation as a function of pre‐ and postfeedback conditions. The postfeedback condition was characterized by increased SCR activity

As shown by the ANOVA, no significant differences in ERs were found for Cond (*F*[1, 19]  = 1.21, *p ≥ *.05, η^2^ = 0.16). In contrast, for RTs, a significant effect was found for Cond (*F*[1, 19]  = 8.12, *p *≤* *.001, η^2^ = 0.32), with increased RTs in postfeedback than prefeedback (Figure 3a).

### 3.2 Autonomic measures

For HR variable, no significant Cond effect was observed (*F*[1, 19]  = 1.02, *p ≥ *.05, η^2^ = 0.17).

For SCR, repeated measures ANOVA showed significant effect for Cond (*F*[1, 19]  = 8.78, *p *≤* *.001, η^2^ = 0.36). Indeed, a general increased SCR activity was found in postfeedback condition than in prefeedback (Figure 3b).

### 3.3 EEG

**Figure 4:**
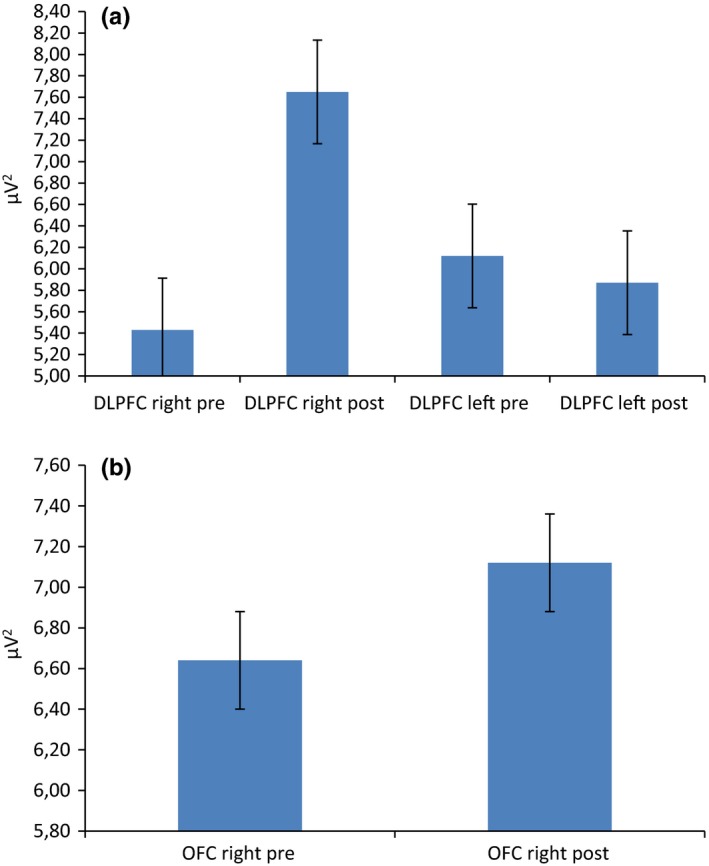
Delta frequency band activity as a function of condition, lateralization, and localization. The postfeedback condition was characterized by a general increased right delta activity over (a) DLPFC and (b) anterior frontal sites

For delta frequency band, repeated measures ANOVA showed significant effect for Cond (*F*[1, 19]  = 8.11, *p *≤* *.001, η^2^ = 0.36) and Cond × Lat × Loc (*F*[1, 92]  = 9.33, *p *≤* *.001, η^2^ = 0.39). Indeed, a general increased delta activity was found in postfeedback condition than in prefeedback. Secondly, as shown by simple effect (contrast analyses for repeated measure ANOVA), delta was increased within the right than left DLPFC area in postfeedback condition (*F*[1, 19]  = 8.90, *p *≤* *.001, ɳ^2^ = 0.35). In addition, right DLPFC activity in postfeedback condition was increased than right DLPFC in prefeedback condition (*F*[1, 19]  = 7.11, *p *≤* *.001, ɳ^2^ = 0.35) (Figure 4a). A significant interaction effect Cond × Lat × Loc was found also over anterior frontal area (*F*[1, 92]  = 9.01, *p *≤* *.001, η^2^ = 0.39) (Figure 4b). Indeed, it was observed a significant increased responsiveness in postfeedback than prefeedback condition within the right DLPFC (*F*[1, 19]  = 7.88, *p *≤* *.001, ɳ^2^ = 0.33). No other effect was significant at the analysis.

**Figure 5:**
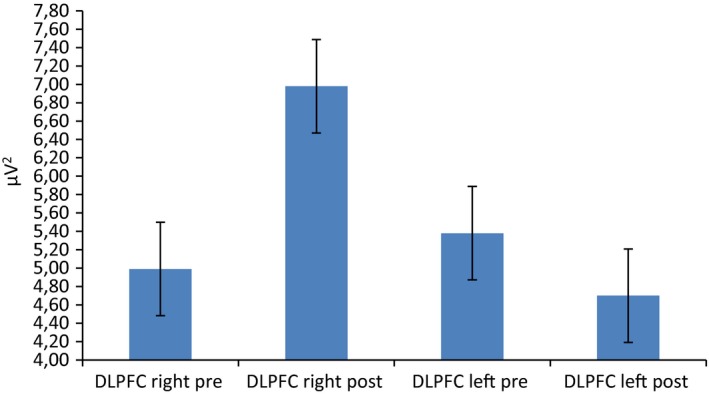
Delta frequency band activity as a function of condition, lateralization, and localization. The postfeedback condition was characterized by a general increased right theta activity over the DLPFC

For theta band, Cond × Lat × Loc interaction effect was significant (*F*[1, 92]  = 8.21, *p *≤* *.001, η^2^ = 0.33). Indeed, as shown by simple effect, theta was increased within the right than left DLPFC area in postfeedback condition (*F*[1, 19]  = 8.11, *p *≤* *.001, ɳ^2^ = 0.32) (Figure 5). In addition, right DLPFC activity in postfeedback condition was increased than right DLPFC in prefeedback condition (*F*[1, 19]  = 6.98, *p *≤* *.001, ɳ^2^ = 0.30).

**Figure 6:**
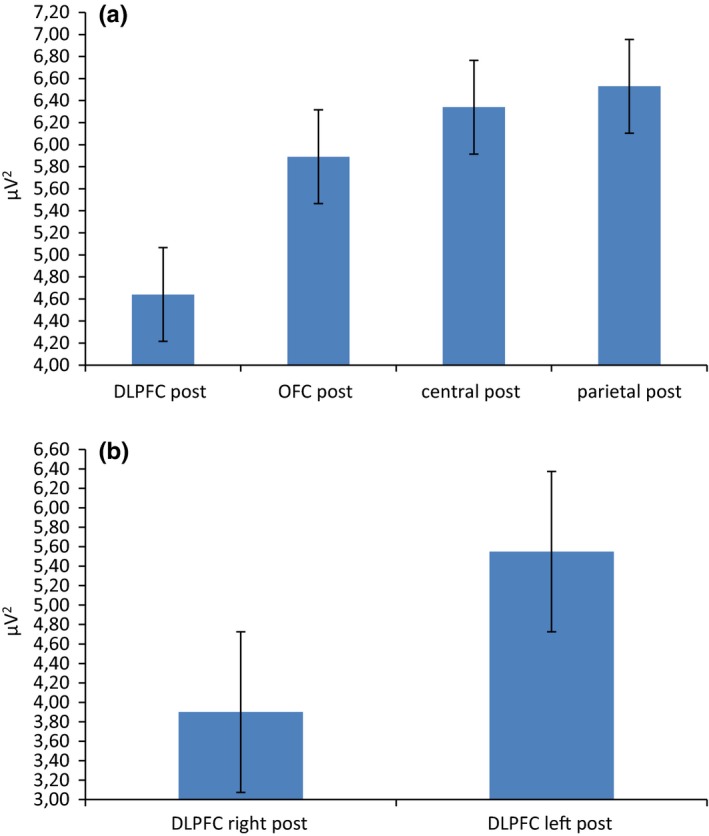
Alpha frequency band activity as a function of condition, lateralization, and localization. (a) A localization effect showing alpha activity decreases activity over the DLPFC with respect to anterior frontal, central, and parietal areas. (b) A lateralization effect showing that the decrease was mainly present within the right, with respect to the left hemisphere

For alpha, Cond × Loc × Lat interaction effect was significant (*F*[1, 92]  = 7.98, *p *≤* *.001, η^2^ = 0.31). Indeed, as shown by post hoc analysis, a decreased alpha activity was found in postfeedback condition within the DLPFC compared to each area (respectively, with orbitofrontal *F*[1, 92]  = 8.43, *p *≤* *.001, η^2^ = 0.32; central *F*[1, 92]  = 9.65, *p *≤* *.001, η^2^ = 0.39; parietal *F*[1, 92]  = 7.12, *p *≤* *.001, η^2^ = 0.30) (Figure 6a). Secondly, alpha was decreased within the right than left DLPFC area in postfeedback condition (*F*[1, 19]  = 6.98, *p *≤* *.001, ɳ^2^ = 0.29) (Figure 6b).

For beta, no significant effects were found.

## 4 DISCUSSION

The present research explored the effects of a negative social feedback during a joint action, considering both the brain and the autonomic contributions, as well as the behavioral performance. Specifically, the brain activation was recorded during a cooperative task which was perceived as failing. A first main effect was related to the systematic impact of the negative feedback on the cortical response, mainly on some specific prefrontal areas (DLPFC and anterior frontal). Secondly, a specific lateralization effect was revealed. Indeed, a significant right‐lateralized activity emerged in postfeedback than in prefeedback condition. Finally, a worse performance in RTs was revealed after the negative social feedback.

The first main effect was related to the increased DLPFC responsiveness after the subjects received their negative feedback. Indeed, we observed a general increased DLPFC activity in the case of a negative condition compared to prefeedback. Such result could be in line with the suggested hypothesis about the need of higher cognitive resources associated with the representation of a negative feedback, with subsequent increased cortical activity (Decety et al., 2004; Gallagher & Frith, 2003). A dysfunctional strategy, even if in a cooperative context, may elicit higher cognitive efforts to update and adjust the joint action. As such, this condition may involve an increase in the cognitive load related to the need of modifying their own strategy, to perform a more efficient cognitive plan, and to include new behavioral possibilities. In addition, previous results revealed that prefrontal areas have a key role in social status regulation and joint actions (De Vico Fallani et al., 2010; Haruno & Kawato, 2009; Karafin, Tranel, & Adolphs, 2004; Suzuki et al., 2011).

In the present research, we found a similar effect, with a significant increased DLPFC activity during a negatively reinforced joint action. This prefrontal area is thought to be involved in social perception, especially when a hierarchy is represented, involving comparisons both across species and human social groups. Therefore, we could assume that this area is responsible for specialized mechanisms to perceive joint actions.

However, this effect was not generalized to each frequency band; indeed, we observed a specific higher responsiveness in low‐frequency band to negative feedback. Indeed, both theta and delta showed increased synchronization after the external feedback, as well as for alpha band although in the opposite direction (decreased alpha synchronization in postfeedback condition). As for the specific contribution of some frequency bands (mainly alpha and theta, more than delta and beta) that we found to be relevant to explain the cortical activation, we may suggest that, on the one hand, alpha may function as an index of brain activation. Indeed, it was found that alpha decreasing may be considered a valid measure of brain activation, and it was largely applied to find distinct responsiveness by specific brain areas to different cognitive or emotional tasks (Balconi & Mazza, 2010; Harmon‐Jones & Allen, 1998). In this case, a general decreased alpha activity in DLPFC may suggest its crucial role for social tasks such as cooperation. On the other hand, theta and delta modulations were previously considered as a specific marker of motivational and emotional components, as well as of the emotional salience of the task, and of the subjects' engagement.

Indeed, it was shown that event‐related theta band power responds to prolonged visual emotional stimulation (Balconi & Vanutelli, 2016; Balconi et al., 2015; Knyazev, 2007; Paré, 2003) in case of a coordinated response indicating alertness and readiness. Specifically, it was shown that the attentional function of theta is derived by the frontal activation, with the probable generators lying in corticohippocampal and frontolimbic structures (Başar, 1999; Karakaş, Erzengin, & Başar, 2000). Also, it has been shown that theta oscillations are involved in memory and emotional regulation (Knyazev, 2007). In some studies, theta power has also been shown to increase when goal conflicts are experienced (Moore, Gale, Morris, & Forrester, 2006; Neo, Thurlow, & McNaughton, 2011; Savostyanov et al., 2009). However, in the present context, we may suggest that theta may preferentially function as a marker of frustration from an unattended feedback of ineffective cooperation and of the negativity of the interpersonal outcomes, as indicated by its sensitivity to the negative feedback. In fact, it has been hypothesized that theta frequency range could be involved in implicit social processing (Yun, Watanabe, & Shimojo, 2012). In fact, besides previous research underlying the role of theta frequency in signaling strategic control and conflict monitoring in social contexts (Billeke, Zamorano, Cosmelli, & Aboitiz, 2013; Cristofori et al., 2013) and the attentive significance of emotional situations (Balconi & Pozzoli, 2009; Balconi et al., 2009a; Başar, 1999), other important findings suggested the involvement of such frequency band during empathic processes, such as empathy for pain (Mu, Fan, Mao, & Han, 2008). For example, Knyazev and colleagues (Knyazev, Slobodskoj‐Plusnin, & Bocharov, 2009) found that theta synchronization is stronger in high sensitive subjects than detached ones. Moreover, Jausovec and colleagues (Jaušovec, Jaušovec, & Gerlič, 2001) found that changes in theta occurring in relation to emotional clips could distinguish among subjects with low versus high scores on emotional intelligence. A similar effect was also present in the form of delta modulation: As already described by Knyazev (Knyazev, 2007), its modulation depends on the activity of motivational systems and can be sensitive to salience detection. Also, stronger delta synchronization was found during the presentation of emotional than neutral stimuli (Knyazev et al., 2009).

A further significant effect is related to the increased right anterior frontal cortex activity in response to negative feedback. A possible interpretation of this result is related to the functional meaning of this area for the cooperative situations. In fact, its role during social cooperative joint actions has already been underlined (Cui et al., 2012), suggesting that this area is involved in goal‐oriented actions such as complex interactive movements and social decision making (Liu et al., 2015). Also, it was related to the voluntary suppression of arousal elicited by emotional stimuli (Cuthbert, Schupp, Bradley, Birbaumer, & Lang, 2000). This is in line with the effect obtained in the present research related to the increased emotional involvement after the negative frustrating feedback.

In addition, a specific hemispheric lateralization was found, with a significant increased activation over the right DLPFC and anterior frontal compared to the left one. This result may be more deeply explained based on the emotional impact hypothesis, which underlined the negative significance of an unsuccessful feedback (Balconi et al., 2012). At this regard, we may consider the increased right PFC responsiveness as a possible marker reflecting the reduction of self‐perceived effectiveness and good performance. Indeed, as previously observed, the frontal cortical asymmetry in favor of the right hemisphere is associated with withdrawal motivation in opposition to approach motivation (Balconi & Mazza, 2010; Davidson, 1993; Harmon‐Jones, Gable, & Peterson, 2010; Jackson et al., 2003; Koslow, Mendes, Pajtas, & Pizzagalli, 2013; Urry et al., 2004). Therefore, we may explain these results taking also into account some previous results on both cooperation and competition, where the DLPFC was found to be mainly activated within the left side in the case of positive cooperation (Balconi & Vanutelli, 2017a) or within the right side in the case of competition (Balconi & Pagani, 2015; Balconi & Vanutelli, 2016). Consequently, activity patterns in the frontal cortices can be regarded to be crucially involved in the processing of emotional conditions which characterize the negative context.

The present effects were also supported by behavioral results: In fact, a significant worse performance, in the form of longer RTs, was found after the negative feedback. Even if we cannot exclude the engagement of higher cognitive effort after the feedback, we may suppose that the worsen performance after the frustrating evaluation may be due to the negative self‐perception and the representation of inefficient joint interactions. These results, in fact, are compatible with findings reported within the tradition of social psychology (Bouffard‐Bouchard, 1990), which highlighted the relation between perceived self‐efficacy and behavioral adjustments (see for example Bandura, 1977).

Finally, this important effect was confirmed by the autonomic modulation, as indexed by SCR. Indeed, it was found that SCR increased after the negative feedback. This effect may be explained taking into account the arousing feature of the negative condition, which was able to modulate the emotional behavior of the subjects. SCR, in fact, is typically used as an objective measure of emotional processing and attention (Damasio, 1994; Frith & Allen, 1983; Öhman & Soares, 1994; Soares & Öhman, 1993). Previous research already found increased SCR for negative compared to positive stimuli (Cobos, Sánchez, García, Nieves Vera, & Vila, 2002; Lane, Reiman, Ahern, Schwartz, & Davidson, 1997; vanOyen Witvliet & Vrana, 2000; Pastor et al., 2008). A possible explanation here is that a negative, frustrating, situation could have triggered greater orienting and attention (Bradley & Lang, 2007; Cuthbert et al., 2000; Lang, Greenwald, Bradley, & Hamm, 1993; Pastor et al., 2008).

Therefore, the present results seem to suggest that the negative cooperative condition generates a significant increasing difficulty in creating a common mental strategy based on a higher workload and most importantly that this behavior (as signaled by EEG and autonomic measures) is due to the emotional negative condition that a frustrating feedback may have created. Thus, we suggest that only the second explanation of the present results, focused on the emotional nature of the social context, could explain the increased lateralization effect found for EEG (more right responsiveness) as a significant prevalence of more negative and avoidance emotions toward the interlocutor, with respect to the cognitive load hypothesis. In fact, it was observed that the right hemisphere is supporting the aversive situations when the subjects have to regulate the conflictual and also divergent goals (Balconi et al., 2012). Therefore, a specific effect like a “negative echo” may be intrinsically related to the failure, with a significant increasing of more withdrawal attitudes.

## 5 CONCLUSIONS

To conclude, frustrating feedback generates behavioral, autonomic, and brain adaptations, being a context which is affected by negative emotions. Some specific areas (mainly the right DLPFC and anterior frontal areas) appeared to be highly implicated as a marker of this social negative effect, where subjects had to adjust their strategies and to manage negative feelings linked to the ineffective performance. The social relevance of this negative feedback and the emotional impact of this unpleasant condition could make the cooperation less “cooperative” and more similar to a “frustrating” condition.

Some limitations may be suggested for the present research. Firstly, sample size is limited and should be extended in future research. Secondly, the implementation of alternative games, which may more directly represent ecological conditions of cooperation, could be considered. Moreover, a wider spatial analysis should be conducted to explore more extensively the whole cortical map during joint cooperative behavior. Finally, future studies could also consider brain‐to‐brain or body‐to‐body coupling analyses (hyperscanning paradigm) to assess whether and how the strength of neural and peripheral synchronization between two interacting subjects change throughout the different conditions presented in the task. These issues was partially addressed in previous research about competition (Balconi & Vanutelli, 2017c) and cooperation (Balconi, Gatti, & Vanutelli, 2017; Balconi & Vanutelli, 2017a) with functional near‐infrared spectroscopy (fNIRS). However, they should be supported in the future by other neural (EEG, temporal features) and peripheral measures.

## CONFLICT OF INTEREST

No conflict of interests to be declared for each author.

## COMPLIANCE WITH ETHICAL STANDARDS

All procedures performed in studies involving human participants were in accordance with the ethical standards of the institutional and/or national research committee and with the 1964 Helsinki declaration and its later amendments or comparable ethical standards. Informed consent was obtained from all individual participants included in the study.

## AUTHORS' CONTRIBUTIONS

Michela Balconi planned the research, supervised the experimental phase, applied the statistical analysis, discussed the data, and wrote the draft. Laura Gatti executed the experiment, applied the statistical analysis, discussed the data, and wrote the draft. Maria Elide Vanutelli executed the experiment, applied the statistical analysis, discussed the data, and wrote the draft.

## SIGNIFICANCE STATEMENT

The present experiment explored neurophysiological component (EEG and autonomic activity) and behavioral response in joined actions during a cooperative task which was inefficient (bad performance). The effects of the negative social feedback in brain and behavior response were analyzed. A specific pattern of brain activation involving the right dorsolateral prefrontal cortex (DLPFC) was observed during inefficient performance. The DLPFC showed increased activity after the feedback (mainly within delta, theta, and alpha frequency bands), compatible with the need of higher cognitive effort. Right‐lateralized effect could be interpreted as increased emotional discomfort and disengagement from goal‐oriented social mechanisms elicited by the negative evaluation.

## References

[brb3902-bib-0001] Amrhein, C. , Mühlberger, A. , Pauli, P. , & Wiedemann, G. (2004). Modulation of event‐related brain potentials during affective picture processing: A complement to startle reflex and skin conductance response? International Journal of Psychophysiology, 54, 231–240. https://doi.org/10.1016/j.ijpsycho.2004.05.009 15331214 10.1016/j.ijpsycho.2004.05.009

[brb3902-bib-0002] Babiloni, F. , Cincotti, F. , Mattia, D. , De Vico Fallani, F. , Tocci, A. , Bianchi, L. , … Astolfi, L. (2007). High resolution EEG hyperscanning during a card game. Eng. Med. Biol. Soc. 2007. EMBS 2007. 29th Annu. Int. Conf. IEEE 4957–4960. https://doi.org/10.1109/iembs.2007.4353453 10.1109/IEMBS.2007.4353453 18003119

[brb3902-bib-0003] Baker, J. M. , Liu, N. , Cui, X. , Vrticka, P. , Saggar, M. , Hosseini, S. M. H. , & Reiss, A. L. (2016). Sex differences in neural and behavioral signatures of cooperation revealed by fNIRS hyperscanning. Scientific Reports, 6, 26492 https://doi.org/10.1038/srep26492 27270754 10.1038/srep26492 PMC4897646

[brb3902-bib-0004] Balconi, M. , & Bortolotti, A. (2012). Resonance mechanism in empathic behavior. BEES, BIS/BAS and psychophysiological contribution. Physiology & Behavior, 105, 298–304. https://doi.org/10.1016/j.physbeh.2011.08.002 21843542 10.1016/j.physbeh.2011.08.002

[brb3902-bib-0005] Balconi, M. , & Bortolotti, A. (2014). Self‐report, personality and autonomic system modulation in response to empathic conflictual versus non conflictual situation. Cognition and Emotion, 28, 153–162. https://doi.org/10.1080/02699931.2013.805685 23768127 10.1080/02699931.2013.805685

[brb3902-bib-0006] Balconi, M. , Bortolotti, A. , & Gonzaga, L. (2011). Emotional face recognition, EMG response, and medial prefrontal activity in empathic behaviour. Neuroscience Research, 71, 251–259. https://doi.org/10.1016/j.neures.2011.07.1833 21840350 10.1016/j.neures.2011.07.1833

[brb3902-bib-0007] Balconi, M. , Brambilla, E. , & Falbo, L. (2009a). BIS/BAS, cortical oscillations and coherence in response to emotional cues. Brain Research Bulletin, 80, 151–157. https://doi.org/10.1016/j.brainresbull.2009.07.001 19591907 10.1016/j.brainresbull.2009.07.001

[brb3902-bib-0008] Balconi, M. , Brambilla, E. , & Falbo, L. (2009b). Appetitive vs. defensive responses to emotional cues. Autonomic measures and brain oscillation modulation. Brain Research, 1296, 72–84. https://doi.org/10.1016/j.brainres.2009.08.056 19703433 10.1016/j.brainres.2009.08.056

[brb3902-bib-0009] Balconi, M. , & Canavesio, Y. (2013). Emotional contagion and trait empathy in prosocial behavior in young people: The contribution of autonomic (facial feedback) and balanced emotional empathy scale (BEES) measures. Journal of Clinical and Experimental Neuropsychology, 35, 41–48. https://doi.org/10.1080/13803395.2012.742492 23157445 10.1080/13803395.2012.742492

[brb3902-bib-0010] Balconi, M. , & Canavesio, Y. (2014). High‐frequency rTMS on DLPFC increases prosocial attitude in case of decision to support people. Social Neuroscience, 9, 82–93. https://doi.org/10.1080/17470919.2013.861361 24279315 10.1080/17470919.2013.861361

[brb3902-bib-0011] Balconi, M. , Falbo, L. , & Conte, V. A. (2012). BIS and BAS correlates with psychophysiological and cortical response systems during aversive and appetitive emotional stimuli processing. Motivation and Emotion, 36, 218–231. https://doi.org/10.1007/s11031-011-9244-7

[brb3902-bib-0012] Balconi, M. , Gatti, L. , & Vanutelli, M. E. (2017). When cooperation goes wrong: Brain and behavioural correlates of ineffective joint strategies in dyads. International Journal of Neuroscience, 128, 155–166. https://doi.org/10.1080/00207454.2017.1379519 28914554 10.1080/00207454.2017.1379519

[brb3902-bib-0013] Balconi, M. , Grippa, E. , & Vanutelli, M. E. (2015). What hemodynamic (fNIRS), electrophysiological (EEG) and autonomic integrated measures can tell us about emotional processing. Brain and Cognition, 95, 67–76. https://doi.org/10.1016/j.bandc.2015.02.001 25721430 10.1016/j.bandc.2015.02.001

[brb3902-bib-0014] Balconi, M. , & Mazza, G. (2009). Brain oscillations and BIS/BAS (behavioral inhibition/activation system) effects on processing masked emotional cues. ERS/ERD and coherence measures of alpha band. International Journal of Psychophysiology, 74, 158–165. https://doi.org/10.1016/j.ijpsycho.2009.08.006 19709636 10.1016/j.ijpsycho.2009.08.006

[brb3902-bib-0015] Balconi, M. , & Mazza, G. (2010). Lateralisation effect in comprehension of emotional facial expression: A comparison between EEG alpha band power and behavioural inhibition (BIS) and activation (BAS) systems. Laterality, 15, 361–384. https://doi.org/10.1080/13576500902886056 19536685 10.1080/13576500902886056

[brb3902-bib-0016] Balconi, M. , & Pagani, S. (2014). Personality correlates (BAS‐BIS), self‐perception of social ranking, and cortical (alpha frequency band) modulation in peer‐group comparison. Physiology & Behavior, 133C, 207–215. https://doi.org/10.1016/j.physbeh.2014.05.043 10.1016/j.physbeh.2014.05.043 24907694

[brb3902-bib-0017] Balconi, M. , & Pagani, S. (2015). Social hierarchies and emotions: Cortical prefrontal activity, facial feedback (EMG), and cognitive performance in a dynamic interaction. Social Neuroscience, 10, 166–178. https://doi.org/10.1080/17470919.2014.977403 25372808 10.1080/17470919.2014.977403

[brb3902-bib-0018] Balconi, M. , & Pozzoli, U. (2008). Event‐related oscillations (ERO) and event‐related potentials (ERP) in emotional face recognition. International Journal of Neuroscience, 118, 1412–1424. https://doi.org/10.1080/00207450601047119 18788026 10.1080/00207450601047119

[brb3902-bib-0019] Balconi, M. , & Pozzoli, U. (2009). Arousal effect on emotional face comprehension. Frequency band changes in different time intervals. Physiology & Behavior, 97, 455–462. https://doi.org/10.1016/j.physbeh.2009.03.023 19341748 10.1016/j.physbeh.2009.03.023

[brb3902-bib-0020] Balconi, M. , & Vanutelli, M. E. (2015). Emotions and BIS/BAS components affect brain activity (ERPs and fNIRS) in observing intra‐species and inter‐species interactions. Brain Imaging and Behavior, 10, 750–760. https://doi.org/10.1007/s11682-015-9443-z 10.1007/s11682-015-9443-z 26319406

[brb3902-bib-0021] Balconi, M. , & Vanutelli, M. E. (2016). Competition in the brain. The contribution of EEG and fNIRS modulation and personality effects in social ranking. Frontiers in Psychology, 7, 1587 https://doi.org/10.3389/fpsyg.2016.01587 27790181 10.3389/fpsyg.2016.01587 PMC5062540

[brb3902-bib-0022] Balconi, M. , & Vanutelli, M. E. (2017a). Brains in competition: Improved cognitive performance and inter‐brain coupling by hyperscanning paradigm with functional Near‐Infrared Spectroscopy. Frontiers in Behavioural Neurosciences, 11, 163 https://doi.org/10.3389/fnbeh.2017.00163 10.3389/fnbeh.2017.00163 PMC5583169 28912697

[brb3902-bib-0023] Balconi, M. , & Vanutelli, M. E. (2017b). Interbrains cooperation: Hyperscanning and self‐perception in joint actions. Journal of Clinical and Experimental Neuropsychology, 13, 1–14. https://doi.org/10.1080/13803395.2016.1253666 10.1080/13803395.2016.1253666 27841088

[brb3902-bib-0024] Balconi, M. , & Vanutelli, M. E. (2017c). Brains in competition. Hyperscanning and cognitive performance in joint‐actions. Frontiers in Behavioural Neurosciences. https://doi.org/10.3389/fnbeh.2017.00163

[brb3902-bib-0025] Bandura, A. (1977). Self‐efficacy: Toward a unifying theory of behavioral change. Psychological Review, 84, 191 https://doi.org/10.1037/0033-295X.84.2.191 847061 10.1037//0033-295x.84.2.191

[brb3902-bib-0026] Başar, E. (1999). Brain function and oscillations: integrative brain function neurophysiology and cognitive processes. Springer, Berlin. https://doi.org/10.1007/978-3-642-59893-7

[brb3902-bib-0027] Beck, A. T. , Steer, R. A. , & Brown, G. K. (1996). Manual for the beck depression inventory – II. San Antonio, TX: Psychological Corporation.

[brb3902-bib-0028] Bekkedal, M. Y. V. , Rossi, J. , & Panksepp, J. (2011). Human brain EEG indices of emotions: Delineating responses to affective vocalizations by measuring frontal theta event‐related synchronization. Neuroscience and Biobehavioral Reviews, 35, 1959–1970. https://doi.org/10.1016/j.neubiorev.2011.05.001 21596060 10.1016/j.neubiorev.2011.05.001

[brb3902-bib-0029] Billeke, P. , Zamorano, F. , Cosmelli, D. , & Aboitiz, F. (2013). Oscillatory brain activity correlates with risk perception and predicts social decisions. Cerebral Cortex, 23, 2872–2883. https://doi.org/10.1093/cercor/bhs269 22941720 10.1093/cercor/bhs269

[brb3902-bib-0030] Bouffard‐Bouchard, T. (1990). Influence of self‐efficacy on performance in a cognitive task. Journal of Social Psychology, 130, 353–363. https://doi.org/10.1080/00224545.1990.9924591

[brb3902-bib-0031] Bradley, M. M. , & Lang, P. J. (2007). The International Affective Picture System (IAPS) in the study of emotion and attention In Coan J. A., & Allen J. J. B. (Eds.), Handbook of emotion elicitation and assessment (pp. 39–46). New York, NY: Oxford Univ Press.

[brb3902-bib-0032] Chiao, J. Y. , Adams, R. B. J. , Tse, P. U. , Lowenthal, L. , Richeson, J. A. , & Ambady, N. (2009). Knowing Who's Boss: fMRI and ERP investigations of social dominance perception. Group Processes & Intergroup Relations, 11, 201–214. https://doi.org/10.1177/1368430207088038 10.1177/1368430207088038 PMC2773146 19893753

[brb3902-bib-0033] Chung, D. , Yun, K. , & Jeong, J. (2008). Neural Mechanisms of Free‐riding and Cooperation in a Public Goods Game: An EEG Hyperscanning Study. Proc. 6th Int. Conf. Cogn. Sci. 2–5.

[brb3902-bib-0034] Chung, D. , Yun, K. , & Jeong, J. (2015). Decoding covert motivations of free riding and cooperation from multi‐feature pattern analysis of EEG signals. Social Cognitive and Affective Neuroscience, 10, 1210–1218. https://doi.org/10.1093/scan/nsv006 25688097 10.1093/scan/nsv006 PMC4560941

[brb3902-bib-0035] Cobos, P. , Sánchez, M. , García, C. , Nieves Vera, M. , & Vila, J. (2002). Revisiting the James versus Cannon debate on emotion: Startle and autonomic modulation in patients with spinal cord injuries. Biological Psychology, 61, 251–269. https://doi.org/10.1016/s0301-0511(02)00061-3 12406609 10.1016/s0301-0511(02)00061-3

[brb3902-bib-0036] Cristofori, I. , Moretti, L. , Harquel, S. , Posada, A. , Deiana, G. , Isnard, J. , … Sirigu, A. (2013). Theta signal as the neural signature of social exclusion. Cerebral Cortex, 23, 2437–2447. https://doi.org/10.1093/cercor/bhs236 22875860 10.1093/cercor/bhs236

[brb3902-bib-0037] Cui, X. , Bryant, D. M. , & Reiss, A. L. (2012). NIRS‐based hyperscanning reveals increased interpersonal coherence in superior frontal cortex during cooperation. NeuroImage, 59, 2430–2437. https://doi.org/10.1016/j.neuroimage.2011.09.003 21933717 10.1016/j.neuroimage.2011.09.003 PMC3254802

[brb3902-bib-0038] Cuthbert, B. N. , Schupp, H. T. , Bradley, M. M. , Birbaumer, N. , & Lang, P. J. (2000). Brain potentials in affective picture processing: Covariation with autonomic arousal and affective report. Biological Psychology, 52, 95–111. https://doi.org/10.1016/S0301-0511(99)00044-7 10699350 10.1016/s0301-0511(99)00044-7

[brb3902-bib-0039] Damasio, A. R. (1994). Descartes' error: Emotion, rationality and the human brain. NY: Avon.

[brb3902-bib-0040] Davidson, R. J. (1992). Emotion and affective style: Hemispheric substrates. Psychological Science, 3, 39–43. https://doi.org/10.1111/j.1467-9280.1992.tb00254.x

[brb3902-bib-0041] Davidson, R. J. (1993). Cerebral asymmetry and emotion: Conceptual and methodological conundrums. Cognition and Emotion, 7, 115–138. https://doi.org/10.1080/02699939308409180

[brb3902-bib-0042] Davidson, R. J. (1998). Anterior electrophysiological asymmetries, emotion, and depression: Conceptual and methodological conundrums. Psychophysiology, 35, 607–614. https://doi.org/10.1017/S0048577298000134 9715104 10.1017/s0048577298000134

[brb3902-bib-0043] Davidson, R. J. (2004). What does the prefrontal cortex “do” in affect: Perspectives on frontal EEG asymmetry research. Biological Psychology, 67, 219–234. https://doi.org/10.1016/j.biopsycho.2004.03.008 15130532 10.1016/j.biopsycho.2004.03.008

[brb3902-bib-0044] De Vico Fallani, F. , Nicosia, V. , Sinatra, R. , Astolfi, L. , Cincotti, F. , Mattia, D. , … Babiloni, F. (2010). Defecting or not defecting: How to “read” human behavior during cooperative games by EEG measurements. PLoS ONE, 5, e14187 https://doi.org/10.1371/journal.pone.0014187 21152069 10.1371/journal.pone.0014187 PMC2995728

[brb3902-bib-0045] Decety, J. , Jackson, P. L. , Sommerville, J. A. , Chaminade, T. , & Meltzoff, A. N. (2004). The neural bases of cooperation and competition: An fMRI investigation. NeuroImage, 23, 744–751. https://doi.org/10.1016/j.neuroimage.2004.05.025 15488424 10.1016/j.neuroimage.2004.05.025 PMC3640982

[brb3902-bib-0046] Frith, C. D. , & Allen, H. A. (1983). The skin conductance orienting response as an index of attention. Biological Psychology, 17, 27–39. https://doi.org/10.1016/0301-0511(83)90064-9 6626635 10.1016/0301-0511(83)90064-9

[brb3902-bib-0047] Funane, T. , Kiguchi, M. , Atsumori, H. , Sato, H. , Kubota, K. , & Koizumi, H. (2011). Synchronous activity of two people's prefrontal cortices during a cooperative task measured by simultaneous near‐infrared spectroscopy. Journal of Biomedical Optics, 16, 77011 https://doi.org/10.1117/1.3602853 10.1117/1.3602853 21806291

[brb3902-bib-0048] Furmark, T. , Fischer, H. , Wik, G. , Larsson, M. , & Fredrikson, M. (1997). The amygdala and individual differences in human fear conditioning. NeuroReport, 8, 3957–3960. https://doi.org/10.1097/00001756-199712220-00021 9462473 10.1097/00001756-199712220-00021

[brb3902-bib-0049] Gallagher, H. L. , & Frith, C. D. (2003). Functional imaging of “theory of mind”. Trends in Cognitive Sciences, 7, 77–83. https://doi.org/10.1016/S1364-6613(02)00025-6 12584026 10.1016/s1364-6613(02)00025-6

[brb3902-bib-0050] Goldman, M. , Stockbauer, J. W. , & McAuliffe, T. G. (1977). Intergroup and intragroup competition and cooperation. Journal of Experimental Social Psychology, 113, 81–88. https://doi.org/10.1016/0022-1031(77)90015-4

[brb3902-bib-0051] Harmon‐Jones, E. (2004). On the relationship of frontal brain activity and anger: Examining the role of attitude toward anger. Cognition and Emotion, 18, 337–361. https://doi.org/10.1080/02699930341000059

[brb3902-bib-0052] Harmon‐Jones, E. , & Allen, J. J. B. (1998). Anger and frontal brain activity: EEG asymmetry consistent with approach motivation despite negative affective valence. Journal of Personality and Social Psychology, 74, 1310–1316. https://doi.org/10.1037/0022-3514.74.5.1310 9599445 10.1037//0022-3514.74.5.1310

[brb3902-bib-0053] Harmon‐Jones, E. , Gable, P. A. , & Peterson, C. K. (2010). The role of asymmetric frontal cortical activity in emotion‐related phenomena: A review and update. Biological Psychology, 84, 451–462. https://doi.org/10.1016/j.biopsycho.2009.08.010 19733618 10.1016/j.biopsycho.2009.08.010

[brb3902-bib-0054] Haruno, M. , & Kawato, M. (2009). Activity in the superior temporal sulcus highlights learning competence in an interaction game. Journal of Neuroscience, 29, 4542–4547. https://doi.org/10.1523/JNEUROSCI.2707-08.2009 19357279 10.1523/JNEUROSCI.2707-08.2009 PMC6665723

[brb3902-bib-0055] Humphrey, N. K. (1988). The social function of intellect In Byrne R. W., & Whiten A. (Eds.), Machiavellian intelligence: Social expertise and the evolution of intellect in monkeys, apes, and humans (pp. 13–26). Oxford, UK: Clarendon.

[brb3902-bib-0056] Jackson, D. C. , Mueller, C. J. , Dolski, I. , Dalton, K. M. , Nitschke, J. B. , Urry, H. L. , … Davidson, R. J. (2003). Now you feel it, now you don't: Frontal brain electrical asymmetry and individual differences in emotion regulation. Psychological Science, 14, 612–617. https://doi.org/10.1046/j.0956-7976.2003.psci_1473.x 14629694 10.1046/j.0956-7976.2003.psci_1473.x

[brb3902-bib-0057] Jaušovec, N. , Jaušovec, K. , & Gerlič, I. (2001). Differences in event‐related and induced EEG patterns in the theta and alpha frequency bands related to human emotional intelligence. Neuroscience Letters, 311, 93–96. https://doi.org/10.1016/S0304-3940(01)02141-3 11567786 10.1016/s0304-3940(01)02141-3

[brb3902-bib-0058] Jung, T.‐P. , Makeig, S. , Humphries, C. , Lee, T.‐W. , McKeown, M. J. , Iragui, V. , & Sejnowski, T. J. (2000). Removing electroencephalographic artifacts by blind source separation. Psychophysiology, 37, 163–178. https://doi.org/10.1111/1469-8986.3720163 10731767

[brb3902-bib-0059] Karafin, M. S. , Tranel, D. , & Adolphs, R. (2004). Dominance attributions following damage to the ventromedial prefrontal cortex. Journal of Cognitive Neuroscience, 16, 1796–1804. https://doi.org/10.1162/0898929042947856 15701229 10.1162/0898929042947856

[brb3902-bib-0060] Karakaş, S. , Erzengin, Ö. U. , & Başar, E. (2000). The genesis of human event‐related responses explained through the theory of oscillatory neural assemblies. Neuroscience Letters, 285, 45–48. https://doi.org/10.1016/S0304-3940(00)01022-3 10788704 10.1016/s0304-3940(00)01022-3

[brb3902-bib-0061] Knoblich, G. , & Jordan, J. S. (2003). Action coordination in groups and individuals: Learning anticipatory control. Journal of Experimental Psychology. Learning, Memory, and Cognition, 29, 1006–1016. https://doi.org/10.1037/0278-7393.29.5.1006 10.1037/0278-7393.29.5.1006 14516231

[brb3902-bib-0062] Knyazev, G. G. (2007). Motivation, emotion, and their inhibitory control mirrored in brain oscillations. Neuroscience and Biobehavioral Reviews, 31, 377–395. https://doi.org/10.1016/j.neubiorev.2006.10.004 17145079 10.1016/j.neubiorev.2006.10.004

[brb3902-bib-0063] Knyazev, G. G. , Slobodskoj‐Plusnin, J. Y. , & Bocharov, A. V. (2009). Event‐related delta and theta synchronization during explicit and implicit emotion processing. Neuroscience, 164, 1588–1600. https://doi.org/10.1016/j.neuroscience.2009.09.057 19796666 10.1016/j.neuroscience.2009.09.057

[brb3902-bib-0064] Koslow, K. , Mendes, W. B. , Pajtas, P. E. , & Pizzagalli, D. A. (2013). Greater left resting intracortical activity as a buffer to social threat. Psychological Science, 22, 641–649. https://doi.org/10.1177/0956797611403156 10.1177/0956797611403156 PMC3196334 21467550

[brb3902-bib-0065] Krause, L. , Enticott, P. G. , Zangen, A. , & Fitzgerald, P. B. (2012). The role of medial prefrontal cortex in theory of mind: A deep rTMS study. Behavioral Brain Research, 228, 87–90. https://doi.org/10.1016/j.bbr.2011.11.037 10.1016/j.bbr.2011.11.037 22155478

[brb3902-bib-0066] Lane, R. , Reiman, E. M. , Ahern, G. L. , Schwartz, G. E. , & Davidson, R. J. (1997). Neuroanatomical correlates of happiness, sadness, and disgust. American Journal of Psychiatry, 154, 926–933. https://doi.org/10.1176/ajp.154.7.926 9210742 10.1176/ajp.154.7.926

[brb3902-bib-0067] Lang, P. J. , Davis, M. , & Ohman, A. (2000). Fear and anxiety: Animal models and human cognitive psychophysiology. Journal of Affective Disorders, 61, 137–159. https://doi.org/10.1016/S0165-0327(00)00343-8 11163418 10.1016/s0165-0327(00)00343-8

[brb3902-bib-0068] Lang, P. J. , Greenwald, M. K. , Bradley, M. M. , & Hamm, A. O. (1993). Looking at pictures: Affective, facial, visceral, and behavioral reactions. Psychophysiology, 30, 261–273. https://doi.org/10.1111/j.1469-8986.1993.tb03352.x 8497555 10.1111/j.1469-8986.1993.tb03352.x

[brb3902-bib-0069] Leslie, A. (1987). Pretense and representation: The origins of “‘Theory of Mind'”. Psychological Review, 94, 412–426. https://doi.org/10.1037/0033-295X.94.4.412

[brb3902-bib-0070] Levitan, R. , Hasey, G. , & Sloman, L. (2000). Major depression and the involuntary defeat strategy: Biological correlates In Gilbert P., & Sloman L. (Eds.), Subordination and defeat: An evolutionary approach to mood disorders and their therapy (pp. 95–120). Manhaw, NJ: Lawrence Erlbaum Associates.

[brb3902-bib-0071] Liu, T. , Saito, H. , & Oi, M. (2015). Role of the right inferior frontal gyrus in turn‐based cooperation and competition: A near‐infrared spectroscopy study. Brain and Cognition, 99, 17–23. https://doi.org/10.1016/j.bandc.2015.07.001 26189111 10.1016/j.bandc.2015.07.001

[brb3902-bib-0072] Locke, E. A. , Frederick, E. , Lee, C. , & Bobko, P. (1984). Effect of self‐efficacy, goals, and task strategies on task performance. Journal of Applied Psychology, 69, 241–251. https://doi.org/10.1037/0021-9010.69.2.241

[brb3902-bib-0073] Ludwig, K. A. , Miriani, R. M. , Langhals, N. B. , Joseph, M. D. , Anderson, D. J. , & Kipke, D. R. (2009). Using a common average reference to improve cortical neuron recordings from microelectrode arrays. Journal of Neurophysiology, 101, 1679–1689. https://doi.org/10.1152/jn.90989.2008 19109453 10.1152/jn.90989.2008 PMC2666412

[brb3902-bib-0074] Marsh, A. A. , Blair, K. S. , Jones, M. M. , Soliman, N. , & Blair, R. J. R. (2009). Dominance and submission: the ventrolateral prefrontal cortex and responses to status cues. Journal of Cognitive Neuroscience, 21, 713–724. https://doi.org/10.1162/jocn.2009.21052 18578604 10.1162/jocn.2009.21052 PMC2735774

[brb3902-bib-0075] Moore, R. A. , Gale, A. , Morris, P. H. , & Forrester, D. (2006). Theta phase locking across the neocortex reflects cortico‐hippocampal recursive communication during goal conflict resolution. International Journal of Psychophysiology, 60, 260–273. https://doi.org/10.1016/j.ijpsycho.2005.06.003 16168505 10.1016/j.ijpsycho.2005.06.003

[brb3902-bib-0076] Morinaga, K. , Akiyoshi, J. , Matsushita, H. , Ichioka, S. , Tanaka, Y. , Tsuru, J. , & Hanada, H. (2007). Anticipatory anxiety‐induced changes in human lateral prefrontal cortex activity. Biological Psychology, 74, 34–38. https://doi.org/10.1016/j.biopsycho.2006.06.005 16893600 10.1016/j.biopsycho.2006.06.005

[brb3902-bib-0077] Mu, Y. , Fan, Y. , Mao, L. , & Han, S. (2008). Event‐related theta and alpha oscillations mediate empathy for pain. Brain Research, 1234, 128–136. https://doi.org/10.1016/j.brainres.2008.07.113 18718453 10.1016/j.brainres.2008.07.113

[brb3902-bib-0078] Neo, P. S. , Thurlow, J. K. , & McNaughton, N. (2011). Stopping, goal‐conflict, trait anxiety and frontal rhythmic power in the stop signal task. Cognitive, Affective, & Behavioral Neuroscience, 11, 485–493. https://doi.org/10.3758/s13415-011-0046-x 10.3758/s13415-011-0046-x 21647572

[brb3902-bib-0079] Öhman, A. , & Soares, J. J. F. (1994). “Unconscious anxiety”: Phobic responses to masked stimuli. Journal of Abnormal Psychology, 103, 231. 8040492 10.1037//0021-843x.103.2.231

[brb3902-bib-0080] Oostenveld, R. , & Praamstra, P. (2001). The five percent electrode system for high‐resolution EEG and ERP measurements. Clinical Neurophysiology, 112, 713–719. https://doi.org/10.1016/S1388-2457(00)00527-7 11275545 10.1016/s1388-2457(00)00527-7

[brb3902-bib-0081] vanOyen Witvliet, C. , & Vrana, S. R. (2000). Emotional imagery, the visual startle, and covariation bias: An affective matching account. Biological Psychology, 52, 187–204. https://doi.org/10.1016/S0301-0511(00)00027-2 10725563 10.1016/s0301-0511(00)00027-2

[brb3902-bib-0082] Paré, D. (2003). Role of the basolateral amygdala in memory consolidation. Progress in Neurobiology, 70, 409–420. https://doi.org/10.1016/S0301-0082(03)00104-7 14511699 10.1016/s0301-0082(03)00104-7

[brb3902-bib-0083] Pastor, M. C. , Bradley, M. M. , Löw, A. , Versace, F. , Moltó, J. , & Lang, P. J. (2008). Affective picture perception: Emotion, context, and the late positive potential. Brain Research, 1189, 145–151. https://doi.org/10.1016/j.brainres.2007.10.072 18068150 10.1016/j.brainres.2007.10.072 PMC2993239

[brb3902-bib-0084] Roach, B. J. , & Mathalon, D. H. (2008). Event‐related EEG time‐frequency analysis: An overview of measures and an analysis of early gamma band phase locking in schizophrenia. Schizophrenia Bulletin, 34, 907–926. https://doi.org/10.1093/schbul/sbn093 18684772 10.1093/schbul/sbn093 PMC2632478

[brb3902-bib-0085] Sänger, J. , Müller, V. , & Lindenberger, U. (2012). Intra‐ and interbrain synchronization and network properties when playing guitar in duets. Frontiers in Human Neuroscience, 6, 312 https://doi.org/10.3389/fnhum.2012.00312 23226120 10.3389/fnhum.2012.00312 PMC3509332

[brb3902-bib-0086] Sapolsky, R. M. (2004). Social status and health in humans and other animals. Annual Review of Anthropology, 33, 393–418. https://doi.org/10.1146/annurev.anthro.33.070203.144000

[brb3902-bib-0087] Savostyanov, A. N. , Tsai, A. C. , Liou, M. , Levin, E. A. , Juin‐Der, L. , Yuryganov, A. V. , & Knyazev, G. G. (2009). EEG correlates of trait anxiety in the stop‐signal paradigm. Neuroscience Letters, 449, 112–116. https://doi.org/10.1016/j.neulet.2008.10.084 18996169 10.1016/j.neulet.2008.10.084

[brb3902-bib-0088] Sebanz, N. , Knoblich, G. , & Prinz, W. (2003). Representing others' actions: just like one's own? Cognition, 88, B11–B21. https://doi.org/10.1016/S0010-0277(03)00043-X 12804818 10.1016/s0010-0277(03)00043-x

[brb3902-bib-0089] Soares, J. J. F. , & Öhman, A. (1993). Preattentive processing, preparedness and phobias: Effects of instruction on conditioned electrodermal responses to masked and non‐masked fear‐relevant. Behavior Research and Therapy, 31, 87–95. https://doi.org/10.1016/0005-7967(93)90046-W 10.1016/0005-7967(93)90046-w 8417731

[brb3902-bib-0090] Spielberger, C. D. , Gorsuch, R. L. , Lushene, R. E. , Vagg, P. R. , & Jacobs, G. A. (1970). STAI manual for the state‐trait anxiety inventory. Santa Clara, CA: Palo Alto, Consulting Psychologists Press.

[brb3902-bib-0091] Sutton, S. K. , & Davidson, R. J. (1997). Prefrontal brain asymmetry: A biological substrate of the behavioral approach and inhibition systems. Psychological Science, 8, 204–210. https://doi.org/10.1111/j.1467-9280.1997.tb00413.x

[brb3902-bib-0092] Suzuki, S. , Niki, K. , Fujisaki, S. , & Akiyama, E. (2011). Neural basis of conditional cooperation. Social Cognitive and Affective Neuroscience, 6, 338–347. https://doi.org/10.1093/scan/nsq042 20501484 10.1093/scan/nsq042 PMC3110432

[brb3902-bib-0093] Tanida, M. , Katsuyama, M. , & Sakatani, K. (2007). Relation between mental stress‐induced prefrontal cortex activity and skin conditions: A near‐infrared spectroscopy study. Brain Research, 1184, 210–216. https://doi.org/10.1016/j.brainres.2007.09.058 17950258 10.1016/j.brainres.2007.09.058

[brb3902-bib-0094] Tupak, S. V. , Dresler, T. , Guhn, A. , Ehlis, A. C. , Fallgatter, A. J. , Pauli, P. , & Herrmann, M. J. (2014). Implicit emotion regulation in the presence of threat: Neural and autonomic correlates. NeuroImage, 85, 372–379. https://doi.org/10.1016/j.neuroimage.2013.09.066 24096027 10.1016/j.neuroimage.2013.09.066

[brb3902-bib-0095] Tuscan, L. A. , Herbert, J. D. , Forman, E. M. , Juarascio, A. S. , Izzetoglu, M. , & Schultheis, M. (2013). Exploring frontal asymmetry using functional near‐infrared spectroscopy: A preliminary study of the effects of social anxiety during interaction and performance tasks. Brain Imaging and Behavior, 7, 140–153. https://doi.org/10.1007/s11682-012-9206-z 23132684 10.1007/s11682-012-9206-z

[brb3902-bib-0096] Urry, H. L. , Nitschke, J. B. , Dolski, I. , Jackson, D. C. , Dalton, K. M. , Mueller, C. J. , … Davidson, R. J. (2004). Making a life worth living. Neural correlates of well‐being. Psychological Science, 15, 367–372. https://doi.org/10.1111/j.0956-7976.2004.00686.x 15147488 10.1111/j.0956-7976.2004.00686.x

[brb3902-bib-0097] Vanutelli, M. E. , Nandrino, J.‐L. , & Balconi, M. (2016). The boundaries of cooperation: Sharing and coupling from ethology to neuroscience. Neuropsychology Trends, 19, 83–104. https://doi.org/10.7358/neur-2016-019-vanu

[brb3902-bib-0098] Yun, K. , Watanabe, K. , & Shimojo, S. (2012). Interpersonal body and neural synchronization as a marker of implicit social interaction. Scientific Reports, 2, 959 https://doi.org/10.1038/srep00959 23233878 10.1038/srep00959 PMC3518815

